# DroneTank: Planning UAVs’ Flights and Sensors’ Data Transmission under Energy Constraints

**DOI:** 10.3390/s18092913

**Published:** 2018-09-02

**Authors:** Runqun Xiong, Feng Shan

**Affiliations:** School of Computer Science and Engineering, Southeast University, Nanjing 211189, China; shanfeng@seu.edu.cn

**Keywords:** dynamic Programming, water-tank method, Unmanned Aerial Vehicle (UAV), flight planning, transmission scheduling

## Abstract

We consider an Unmanned Aerial Vehicle (UAV, also known as drone) as an aerial sink to travel along a natural landscape or rural industrial linear infrastructure to collect data from deployed sensors. We study a joint schedule problem that involves flight planning for the drone and transmission scheduling for sensors, such that the maximum amount of data can be collected with a limited individual energy budget for the UAV and the sensors, respectively. On one hand, the flight planning decides the flight speed and flight path based on sensor locations, energy budgets, and the transmission schedule. On the other hand, the transmission schedule decides for each sensor when to deliver data and what transmission power to use based on the energy budgets and flight plan. By observing three import optimality properties, we decouple the joint problem into two subproblems: drone flight planning and sensor transmission scheduling. For the first problem, we propose a dynamic programming algorithm to produce the optimal flight planning. For the second problem, with a flight plan as input, we introduce a novel technique (*water-tank*), which together with dynamic programming, is the key to achieve an optimal transmission schedule that maximizes data collection. Simulations show that the separately determined flight plan and transmission schedule are near-optimal for the original joint problem.

## 1. Introduction

Unmanned Aerial Vehicles (UAVs, also known as drones) are gaining popularity for their potential applications in many scenarios, such as smart grids [1], smart cities [2], precision farming [3], and emergency wireless network access [4]. Often times, UAVs are considered as mobile aerial sinks to quickly collect data from deployed ground sensors. This is because UAVs can reach remote fields with ease and provide more reliable collection methods, compared to traditional wireless relay methods. Some examples of such application scenarios include the power grid transmission tower information collection—where transmission tower structural health monitoring sensors are deployed in rural areas, and using UAVs is more efficient; and large coastal cities safety monitoring—where hydrology sensors are deployed in rivers and nearby coasts for safety monitoring, which requires sensed data to be collected in a timely and periodic manner. In the academic field, there has been a growing interest recently in employing the UAV as a mobile data collector for the ground sensor nodes in wireless sensor networks [5,6,7,8,9,10,11]. However, some of them considered a scenario where a UAV collects data from a set of sensors on a straight line [5,6,7,8], which has limitations in real-world applications; moreover, some of them applied the convex optimization techniques to solve the UAVs’ trajectory optimization problems [9,10,11], such solutions require high time complexity to archive high accuracy using iterative method.

**Figure 1:**
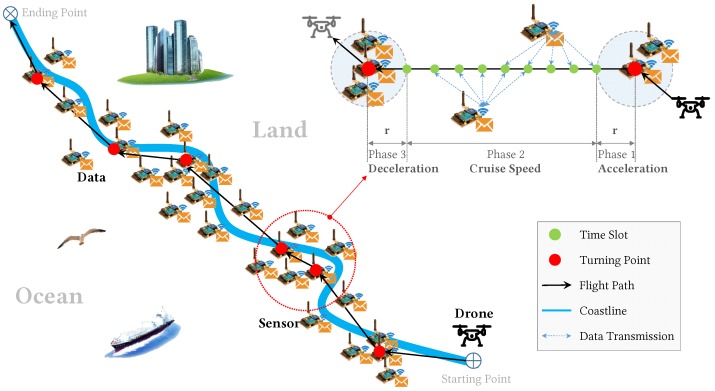
Illustration of a drone flies along a coastline to collect data from deployed sensors. By mainstream open-source or commercial flight controllers, a flight path consists of a sequence of way-points that the drone visits and makes turn at, so they are also called turning points (red solid points). More turning points means more energy consumption of the drone since more flight time and distance to cover, however, it also means closer the drone can fly to sensors to collect data. Fewer turning points consumes less drone energy, but cost sensors to use higher power to transmit data due to the longer distance. The best trade-off with limited drone and sensors energy must be found.

In this paper, we consider a scenario where a UAV flies over a smooth-curve stripe area to collect data from deployed monitoring sensors. We assume that sensors are deployed in a natural landscape or rural industrial linear infrastructure, such as coasts, rivers, power transmission lines, or oil/gas/water pipes, which is not necessarily in a straight line, as shown in Figure 1. We also assume the sensors are nonuniformly distributed, e.g., sensitive areas use more sensors. The UAV must determine its flight path and flight speed to conduct data collection. Meanwhile, each sensor must determine when to transmit data and how large power to transmit to the drone. Because the drone can recharge after a collection trip and sensors can harvest energy and renew its battery between two trips (using sources from the surrounding environment, such as wind, wave, or solar) [12,13,14,15], the goal of our problem is to maximize data collection in a single trip with given energy budgets for the drone and sensors.

The data collection is mainly affected by transmission time and the wireless transmission rate, while the transmission rate is determined by transmission distance and transmission power. As a result, we must control the drones’ flight path and speed carefully, deciding the transmission time and power for each sensor. This is important, because the closer a drone flies to a sensor, the more data can be delivered consuming the same amount of transmission energy. If a drone flies slowly, it provides more time for sensors to deliver data. Moreover, any distance covered or turn made by the drone consumes energy. Considering the drone’s limited energy budget for a single data-collection trip, and its need to navigate sensors that are not deployed in a straight line or uniformly, designing the flight plan is nontrivial. Meanwhile, a sensor can wait until the drone comes near to begin transmission, thereby saving energy. However, when the drone comes near, it may also come close to other sensors. This results in sensors competing for time slots to deliver data. Then sensors with larger energy budget have advantage since they can transmit data from a larger distance, while small-budget sensors must wait until the drone is near. This requires an added level of planning, though, deciding for each sensor when to transmit, and how much transmission power to use, to maximize data delivery.

This joint schedule problem is quite an undertaking. To solve it, we summarize our main contributions as follows.

- First, we formally define the joint UAV flight planning and sensor transmission scheduling (UfpSts) problem. Using three key observations, we decouple the joint problem into two subproblems: UAV flight planning (UAV-FP) and sensor transmission scheduling (SEN-TS).
- Then, for the UAV-FP problem that determines the flight path and speed, we reduce it to determine the number of turns and turning locations. We then propose a dynamic programming approach to produce an optimal solution.
- Next, with the flight plans given, the SEN-TS problem determines for each sensor the time slot assignment and power control. We introduce a novel technique named *water-tank* to compute for a sensor its transmission power in given time slots. We then design a dynamic programming algorithm that uses *water-tank* as a subroutine to optimally solve the SEN-TS problem.
- Finally, in our simulations, the separately computed flight plan and transmission schedule are compared with the optimal solution for the original joint problem. The simulation results show that our proposed algorithms produce a near-optimal solution.

The remainder of this paper is organized as follows. Section 2 reviews related work. Section 3 presents a formal definition of the UfpSts problem. Section 4 separates it into two subproblems: the UAV-FP problem and the SEN-TS problem. A dynamic programming approach is proposed in Section 5 to optimally solve the UAV-FP problem. Section 6 introduces the *water-tank* technique, then uses a dynamic program to solve the SEN-TS problem. Simulation results are presented in Section 7 and Section 8 concludes this paper and discusses the future work.

## 2. Related Work

There has been a growing interest recently in employing the UAV as a mobile data collector for the ground sensor nodes in wireless sensor networks [5,6,7], but some of them only considered a scenario where a UAV collects data from a set of sensors on a straight line, such as [7]. It has been shown that short-distance line-of-sight (LoS) communication links between UAV and ground terminals can be efficiently exploited in various UAV-enabled wireless networks for performance enhancement by properly designing the UAV’s trajectory [8,9,10,11]. Specifically, Zeng et al. [8,9] rigorously studied the UAV trajectory design for a mobile relaying system and point-to-point energy-efficient system, respectively, where sequential convex optimization techniques are applied to solve the non-convex trajectory optimization problems therein. Though providing a general framework for trajectory optimization in two-dimensional space, the studies in [8,9] only focus on the setup with single UAV and single ground user, and the time complexity of their approaches are insufficient. Authors in [10] studied a general multi-UAV enabled wireless communication system, where multiple UAVs are employed to serve a group of users on the ground and proposed an efficient iterative algorithm for solving it by applying the block coordinate descent and successive convex optimization techniques. Yang et al. [11] studied a ground to UAV wireless communication system, where a UAV was dispatched as a mobile data collector to gather a given amount of data from a fixed ground terminal at known location. Meanwhile, researchers have given significant attention to UAV energy-efficient path planning recently [16,17]. Ahmad et al. [16] designed and planned an energy-efficient flight path by modeling the energy consumption of UAV theoretically. Then they analyzed the energy consumption of a straight flight and modeled flight turns. In contrast, Modares et al. [17] studied flight modeling from the aspect of experiments, where the experiments determined the parameters.

There has also been extensive research on collecting the mobile sink from deployed sensors [18,19,20,21,22,23,24,25,26]. Such sink mobility significantly increases a network’s lifetime and improves network throughput [19,20,21,22,23,24,27,28]. However, because of the mobility, the distance between a sensor and the sink becomes variable, affecting the transmission energy (or power) and rate. Regarding sink mobility, there are three commonly used channel models. In the first channel model, energy consumption is assumed to be proportional to the amount of data transmitted (or received) regardless of the transmission rate, transmission power level, or changing distance between the transmitter and receiver [24,27,28]. In the second channel model, if a fixed transmission power is used at the transmitter side, then, according to the book by Tse et al. [29], the received power at the receiver side decays exponentially with the distance, and the date rate is proportional to the received power. This is referred to as the proportional model, and is assumed by many works [19,20,21,22]. The third channel model is the classic Additive White Gaussian Noise (AWGN) channel, which is more sophisticated, realistic, and widely adopted. In this model, the data rate is related to the power received through a convex function called the Shannon-Hartley formula [29]. The work of Chakrabarti et al. in this area is notable [23].

Recent research has also focused on energy harvesting. For example, Yang and Ulukus [30,31] proposed an algorithm that efficiently uses harvested energy to transmit all the arrived packets in a minimum amount of time under the AWGN model. Shan et al. [32] proposed a method named Truncation that produces an optimal discrete rate scheduling for the packet, with individual deadlines in the energy-harvesting system under the AWGN model. However, this research has not considered how to achieve maximum throughput when the receiver or sink moves (instead of being stationary).

## 3. Formulating the Problem

We assume *n* energy harvesting sensors are deployed nonuniformly, following a natural landscape or rural linear industrial infrastructure (such as a coast, river, power transmission line, or oil/gas/water pipe), to monitor information of interest. These sensors are indexed as $s_{1},s_{2},\ldots ,s_{n}$ following their geographic order. Notation $s_{i}$ is also used to denote the sensor’s location, if no ambiguity arises. The harvested energy by a sensor is stored in its battery. Let $E_{i}\left(1\leq i\leq n\right)$, denote the available energy budget of $s_{i}$ for the current data collection trip (introduced later). As assumed in reference [18], each sensor has sufficient data available to transmit to the sink. Note that our proposed approach easily extends to accommodate instances where each sensor has limited available data. We leave this extension to the reader, so that we can detail the key ideas of our method.

A UAV travels along such a smooth-curve stripe to collection data periodically, so such trips called data collection trips. For ease of presentation and algorithm design, the system time is assumed to be slotted equally with the unit length. Let U denote the flight plan for UAV a trip. Because the time slot is so small, the trajectory of UAV in a slot *i* can be represented by a static position $u_{i}$, called the flight position. Therefore, U can be represented by a sequence of flight positions $u_{1},u_{2},\ldots ,u_{m}$, which imply the flight distance, flight speed, and turns of U. Such factors determine the flight energy consumption E(U). Then, we have the energy constraint for the UAV:

(1) $$E\left(U\right)\leq E_{\mathrm{UAV}},$$

where $E_{\mathrm{UAV}}$ is the energy budget of the UAV for the current collection trip. We introduce the specific function of E(U) in the next section (see Equation (8)).

In time slot *i*, the UAV is at position $u_{i}$, so the distance to sensor $s_{i}$ is denoted as $∥s_{i}-u_{j}∥$, ∀i,j, and the UAV is allowed to receive from only one sensor at each position (in each time slot). So, each sensor needs to decide in which time slots it should transmit data. Let binary variable $a_{ij}$ denote the slot-assignment decision:

(2) $$a_{ij}=\left\{0,1\right\},\forall i,j.$$

Sensor $s_{i}$ transmits data to the UAV in time slot *j* at distance $∥s_{i}-u_{j}∥$, if $a_{ij}=1$. It does not transmit if $a_{ij}=0$. Obviously, at each time slot *j*, at most one sensor is allowed to transmit.

(3) $$\sum_{i=1}^{n}a_{ij}\leq 1,\forall j.$$

After the slot assignment is determined, each sensor needs to control its transmission power. Let $p_{ij}$ denote the transmission power of sensor $s_{i}$ in time slot *j*. Such power cannot exceed the maximum power $p^{max}$ imposed by the hardware, hence

(4) $$0\leq p_{ij}\leq p^{max},\forall i,j.$$

At the same time, all energy consumed in transmission should not exceed the energy budget for sensor *i*, so

(5) $$\sum_{j=1}^{m}a_{ij}p_{ij}\leq E_{i},\forall i.$$

Note that because the time is slotted with the unit length, the transmission power $p_{ij}$ in slot *j* equals its energy consumption.

In Equation (6), the AWGN channel model is adopted so that the relation between transmission power $p_{ij}$, transmission distance $∥s_{i}-u_{j}∥$, and transmission rate $r_{ij}$ follow the Shannon-Hartley formula [29]:

(6) $$r_{ij}=\log\left(1+\frac{p_{ij}}{∥s_{i}-u_{j}{∥}^{\alpha }}\right),$$

where α is called the path loss exponent and usually ranges between 2 and 6.

**Remark** **1.** *Some research papers [18,19,20,21,22] assume $r\propto (p/d^{\alpha })$, which is a simplified model of Equation (6). Some research papers [30,31,32,33,34] adopt r=log(1+p), which is a special case of Equation (6). Chakrabarti et al. [23] adopt the Equation (6)’s model, but assume that p is fixed instead of being adjustable. Our model is the most general model.*

Hence, the total data collected by the UAV in a single trip can be expressed as

(7) $$\sum_{j=1}^{m}\sum_{i=1}^{n}a_{ij}r_{ij}.$$

**Definition** **1.** *(UfpSts Problem). Give a set of n energy harvesting sensors $\left\{s_{i},i=1,2,\ldots ,n\right\}$, the energy budget $E_{i}$ for sensor $s_{i}$ and the energy budget $E_{\mathrm{UAV}}$ for the UAV, the UAV flight planning and Sensor transmission schedule for maximum data collection (UfpSts) problem is to jointly determine UAV flight plan U, sensor transmission schedule, e.g., slot assignment $\left\{a_{ij}\right\}$ and power control $\left\{p_{ij}\right\}$, such that data collection (Equation (7)) is maximized and Equations (1)–(6) are satisfied*

## 4. Problem Decoupling and Subproblems

To simplify algorithm design and analysis, we assume the UAVs either fly at the cruise speed, or slow down to make turns and then speed up to reach the cruise speed again. This is what most mainstream open-source or commercial UAV flight controllers [35] do to provide safe and robust flight control. Hence, we reduce the flight plan problem to ask how many turns are needed and where to make them. After determining all the turning points, then we determine the flight plan—e.g., the flight path and speed. Note that by making a 0-degree turn, the UAV performs a slowdown operation.

Under such an assumption, the flight plan’s energy consumption consists of two parts: the energy consumed in covering the distance and the energy consumed in making turns. By normalization, we assume that each unit of the distance covered costs one unit of drone energy, and each turn costs *C* units of drone energy. Hence, we have

(8) $$E_{U}=∥U∥+C\times turns\left(U\right),$$

where ∥U∥ denotes the total flight distance (at cruise speed) of flight plan U and turns(U) denotes the number of turns (including deceleration and acceleration) in U, and *C* includes deceleration, turning and acceleration energy consumption.

The UfpSts problem in Definition 1 not only considers the determination of flight plan U, but also considers the slot assignment $\left\{a_{ij}\right\}$ and power control $\left\{p_{ij}\right\}$. In other words, UfpSts problem consists of flight planning for the UAV and transmission scheduling for sensors. They are jointly determined, because they affect each other. On the one hand, inappropriate flight planning cause the UAV be far from some sensors such that even the optimal transmission scheduling cost a lot sensor energy to transmit data due to long distance. On the other hand, inappropriate transmission schedule may require a sensor to transmit data when the UAV is still far away instead of wait for it to come closer to improve energy efficient transmission; inappropriate transmission schedule may also require large-energy-budget sensor to delivery at a low power/rate occupying too many time slots and the UAV may have already gone away. In a summary, flight planning for the UAV and transmission scheduling for sensors are highly coupled. It is rather complicated to solve this joint problem directly. So, we divide it into two simpler subproblems, with one focus on UAV flight planning, and the other on sensor transmission scheduling. The key to such decoupling is to clarify the connection between UAV flight planning and sensor transmission scheduling, to maximize data collection. We observed some key properties and the intuition is, as long as the UAV flies closer and slower to sensors, and considers sensors with more energy more important, then there will be good transmission schedules. In this way, the flight planning can focus on satisfying such properties instead of the transmission scheduling for sensors. We thus present three key observations.

**Observations 1** (Fly closer). According to the Shannon-Hartley Theorem of the AWGN channel, when a UAV flies closer to a sensor, the sensor transmits data to the UAV with greater energy efficiency.

**Observations 2** (Fly slower). The slower the UAV flies, the more time for sensors to deliver data, and hence, more data are collected. However, flying close to every sensor and flying slow costs energy, and the UAV has a limited energy budget for the data-collection trip. When conflict occurs, our third observation can guide the trade-off.

**Observations 3** (More energy, more important). If a sensor has a larger energy budget, it can deliver more data to the UAV; so, the energy budget is the most important aspect of flight planning.

Based on these three observations, we first define the UAV flight plan problem, which computes the turning points (and thus the flight plan U) according to $\left\{s_{i}\right\}$ and $\left\{E_{i}\right\}$. Then we define the sensor transmission schedule problem, which computes $\left\{a_{ij}\right\}$ and $\left\{p_{ij}\right\}$ according to previously computed U. The ultimate goal is to maximize data collection.

### 4.1. Planning UAV Flights

Assume the UAV makes a turn only at the position right above a sensor. Then, there are *n* candidate turning points, denoted as $\left\{S_{1},S_{2},\ldots ,S_{n}\right\}$, where position $S_{i}$ is right above sensor $s_{i}$. Assume $\left\{T_{0},T_{1},T_{2},\ldots ,T_{k}\right\}$ are the selected turning points, where is $T_{0}$ and $T_{k}=S_{n}$ are the flight plan’s starting and ending points. We have $\left\{T_{1},T_{2},\ldots ,T_{k}\right\}\subset \left\{S_{1},S_{2},\ldots ,S_{n}\right\}$. Between two adjacent turning points, the UAV takes the straight flight path. Then the turning point set uniquely determines the flight plan U.

Since any U can not satisfy the three observations for all sensors simultaneously, we use error to measure a given U. Each sensor has an error, and the accumulated error is used to judge the given U. We define two types of errors: the turn error and the line error. A sensor either has the turn error or the line error, depending on whether it is close to a turning point.

Because the UAV flies slowly around a turning point, a sensor within *r* distance to turning point $S_{i}$ is associated with it, and is assumed to have the turn error, where *r* is the distance covered for slowing down (or speeding up). The intuition behind is, since UAV has to slow down to make a turn and then speed up, sensors around the turning point can benefit by transmitting to the UAV when it is slow and close. We define the turn error of sensor *k* for $S_{i}$ as $∥S_{i}-s_{k}{∥}^{2}\sqrt{E_{k}}$—e.g., a longer distance leads to greater opportunity for error; and the larger the energy budget, the greater the opportunity for error. Both are consistent with our observations. The sum of the turn error for turning point $S_{i}$ is computed as

(9) $$E_{r}^{T}\left(i\right)=\sum_{k=Rl(i)}^{Rr(i)}{∥S_{i}-s_{k}∥}^{2}\sqrt{E_{k}},$$

where [Rl(i),Rr(i)] are sensors within *r* distance to $S_{i}$. Because the sensors are indexed according to their geographic location, we assume these sensors have continuous indices.

We now define the line error by considering the straight flight path between two adjacent turning points $S_{i}$ to $S_{j}$. Let the line be denoted as $L_{i,j}$. Since some sensors around $L_{i,j}$ have already been assigned the turn error, and only sensors in [Rr(i)+1,Rl(j)−1] are left without assigned errors. We define the line error as follows:

(10) $$E_{r}^{L}\left(i,j\right)=7\sum_{k=Rr(i)+1}^{Rl(j)-1}{∥L_{i,j}-s_{k}∥}^{2}\sqrt{E_{k}},$$

where $∥L_{i,j}-s_{k}{∥}^{2}$ is the squared distance from location $s_{k}$ to the flight line $L_{i,j}$. Similarly, this definition satisfies the observations. Note that, in this equation, the constant 7 increases the line error, because a sensor associated with a turn has greater opportunity to transmit data than one associated with a line.

In a given flight plan U, $\left\{T_{0},T_{1},T_{2},\ldots ,T_{k}\right\}$ are the turning points, so flight plan U’s total error is therefore the sum of all the turn and line errors:

(11) $$E_{r}\left(U\right)=\sum_{i=0}^{k-1}\left(E_{r}^{T}\left(T_{i}\right)+E_{r}^{L}\left(T_{i},T_{i+1}\right)\right).$$

The energy consumption of flight plan U cannot exceed the UAV’s energy budget, so

(12) $$E\left(U\right)=\sum_{i=1}^{k}(C+∥L_{T_{i-1},T_{i}}∥)\leq E_{\mathrm{UAV}}.$$

**Definition** **2.** *(UAV-FP Problem). Given a set of n energy-harvesting sensors $\left\{s_{i}\right\}$, the energy budget $E_{i}$ for sensor $s_{i}$ and the energy budget $E_{\mathrm{UAV}}$ for the UAV, the UAVFlight Planning (UAV-FP) problem aims to ascertain the flight plan U by determining the turning point $T_{i},i=1,2,\ldots ,k$, (and the number k) such that the total error (Equation (11)) is minimized, while Equations (9), (10), and (12) are satisfied.*

### 4.2. Scheduling Sensor Transmission

Once the flight plan U is determined, all the flight positions $\left\{u_{i}|i=1,2,\ldots ,m\right\}$ are determined. Because the flight path is a smooth-curve stripe, we assume each sensor has a set of available slots in which it can transmit data to the UAV. That is to say, only $u_{i}$ that is within a communication range of the sensor is considered available for data delivery. We assume the available slots of sensor $s_{i},1\leq i\leq n$, are consecutive, $\left(b_{i},e_{i}\right]=\left\{slot\;b_{i}+1,slot\;b_{i}+2,\ldots slot\;e_{i}\right\},b_{i}\leq e_{i}$.

**Definition** **3.** *(Slot Assignment). Given a flight plan U, a set of energy renewable sensors, $s_{i},1\leq i\leq n$, and m slots as discussed above, a slot assignment is to assign a set of consecutive slots $\left(\beta_{i},e_{i}\right]$ to each and every sensor $s_{i},1\leq i\leq n$, such that*

*(a)*  *$\left(\beta_{i},e_{i}\right]$ is a subset of available slot sets $\left(b_{i},e_{i}\right]$.*
*(b)*  *Assignments, $(b_{i},e_{i}],1\leq i\leq n$, are mutually disjoint. Without loss of generality, we assume*
  (13) $$0\leq \beta_{1}\leq \varepsilon_{1}\leq \beta_{2}\leq \varepsilon_{2}\leq \ldots \beta_{n}\leq \varepsilon_{n}\leq m.$$

**Definition** **4.** *(Power Control). Given a slot assignment $\left(\beta_{i},e_{i}\right]$ to each sensor $s_{i},1\leq i\leq n$, a power control is to determine the transmission power $p_{ij}$ to be used by sensor $s_{i}$ for slot $j,j=\beta_{i}+1,\beta_{i}+2,\ldots ,\varepsilon_{i}$, where $0\leq p_{ij}\leq p^{max}$. Moreover, the total energy consumption must satisfy*

(14) $$\sum_{j=1}^{m}p_{ij}=\sum_{j=\beta_{i}+1}^{\varepsilon_{i}}p_{ij}\leq E_{i},$$

*where $p_{ij}=0$, if $j\notin (\beta_{i},\varepsilon_{i})$.*

*Obviously, according to the Shannon-Hartley formula, that is, Equation (6), the total amount of data B transmitted to the mobile sink from all n sensors is*

(15) $$B=\sum_{i=1}^{n}\sum_{j=1}^{m}\log\left(1+\frac{p_{ij}}{d_{ij}^{\alpha }}\right),$$

*where $d_{ij}=∥s_{i}-u_{j}∥$.*

**Definition** **5.** *(SEN-TS Problem) Given a flight plan U, energy harvesting sensors $s_{i}$ and sensor energy budget $E_{i},1\leq i\leq n$, the SENsor Transmission Scheduling (SEN-TS) problem is to determine a slot assignment and a power control such that the total amount of transmitted data computed by Equation (15) is maximized.*

## 5. Optimally Solving the UAV-FP Problem

In this section, we focus on the UAV-FP problem, namely to determine the optimal number of turning points and their optimal locations. We use dynamic programming to find the best solution.

Recall that in the optimal solution, a turn centered at $S_{i}$ costs *C* units of UAV energy and generates $E_{r}^{T}\left(i\right)$ turn errors, while a straight flight path between $S_{i}$ and $S_{j}$ costs $d_{i,j}$ units of UAV energy and generates an $E_{r}^{L}\left(i,j\right)$ line error. We group a turning point and its following straight flight path together to form a turn line, and use dynamic programming to determine each turn line of the optimal solution.

Although we do not know what exactly the optimal solution is, one thing is for sure: the last sensor $s_{n}$ belongs to the last turn line, and such a turn line must begin with an earlier sensor. (Because they are fixed, we ignore both the energy costs and turn error of the last turning point at $S_{n}$.) This observation leads us to an interesting subproblem: if we know in the optimal solution that the last turn line starts from $s_{i}$ and ends at $s_{n}$, then we knew the exact energy consumption of this turn line, and hence, we could remove those sensors from consideration and recursively solve the problem on the remaining sensors $s_{1},s_{2},\ldots ,s_{i}$ with a reduced energy budget. We define such a subproblem as $E_{r}\left(e,i\right)$, presenting the optimum solution (the flight plan with the fewest errors) for sensors $s_{1},s_{2},\ldots ,s_{i}$ using UAV energy budget *e*. Then, our observation is that

(16) $$E_{r}\left(E_{UAV},n\right)=E_{r}^{C}\left(i\right)+E_{r}^{L}\left(i,n\right)+E_{r}\left(E_{UAV}-C-d_{i,n},i\right).$$

Using the same observation for the subproblem consisting of sensors $s_{1},s_{2},\ldots ,s_{i}$, we see that to get an optimal value of $E_{r}\left(e,i\right)$, we should find the best way to produce a final turn line covering $s_{j},s_{j+1},\ldots ,s_{i}$, which costs $C+d_{i,j}$ energy and gains an error of $E_{r}^{C}\left(j\right)+E_{r}^{L}\left(j,i\right)$. So, the energy budget for the remaining sensors $s_{1},s_{2},\ldots ,s_{j}$ is reduced to $e-C-d_{i,j}$. We therefore have the following recurrence:

(17) $$E_{r}\left(e,i\right)=\min_{j:Rr(j)<Rl(i)}\left\{E_{r}^{C}\left(j\right)+E_{r}^{L}\left(j,i\right)+E_{r}\left(e-C-d_{i,j},j\right)\right\}.$$

Recall that [Rl(i),Rr(i)] are sensors within *r* distance to $S_{i}$. Note that we assume the UAV does not make two turns close to each other, so we have Rr(j)<Rl(i), for j<i. In addition to the value assignment for $E_{r}\left(e,i\right)$, the following two special cases are supplementary: when i=1, there is only one sensor to cover, so there is no error, $E_{r}\left(e,i\right)=0$; when $e<d_{1,i}+C$, the energy budget does not support even the simplest flight plan, so $E_{r}\left(e,i\right)=\infty$. Thus, we have the following cases:

(18) $$E_{r}\left(e,i\right)=\left\{\begin{array}{cc} 0 & \mathrm{if}\;i=1\\ \infty & \mathrm{if}\;e<d_{1,i}+C \end{array}\right.$$

The detailed dynamic programming pseudocode for solving the UAV-FP problem is presented in Algorithm 1 (UAV-FP-SVR). Note that, for computation purposes, we round all variables $d_{i,j},C,E_{UAV}$ to integers.

**Algorithm 1:** UAV-FP-SVR

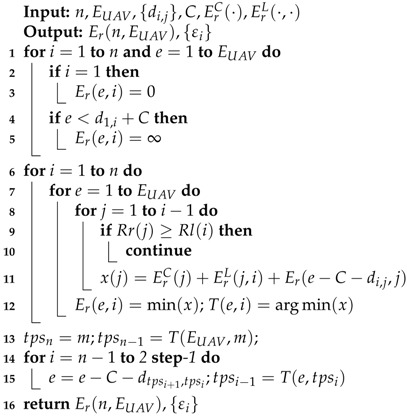



## 6. Optimally Solving the SEN-TS Problem

Here, we present an optimal algorithm to solve the SEN-TS problem with the given flight plans. Specifically, we introduce a novel technique named *water-tank*, which uses dynamic programming to solve the SEN-TS problem.

### 6.1. The Single-Sensor SEN-TS-1 Problem

We first study a simplified SEN-TS problem, where only one sensor is deployed along the flight path (the SEN-TS-1 problem). The SEN-TS-1 problem is simple enough and can be formulized as a convex program. Solving the SEN-TS-1 problem by solving the convex program is not an interesting method we wish to present. However, by analyzing this convex program’s optimality property, we discover an interesting and efficient method.

**Figure 2:**
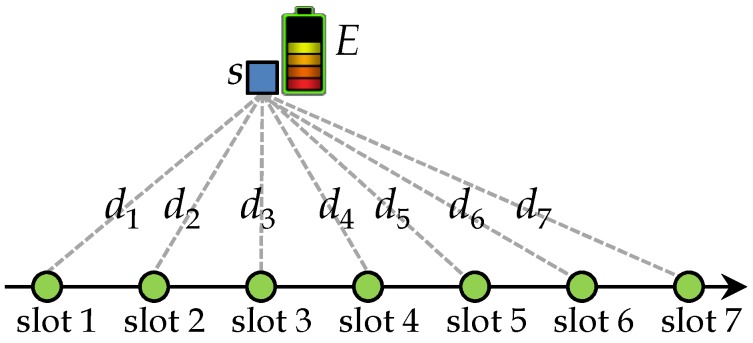
An example of the SEN-TS-1 problem, where the available slots are (0,7]. Each circle represents the flight position in each time slot and dashed lines show the distance between this position and sensor *s*. The goal is to determine the optimal transmission power *p* in each slot, such that the data transmitted by *s* (collected by sink *S*) is maximized with the battery’s limited energy *E*.

In the SEN-TS-1 problem, let $d_{j}$ be the distance between the only sensor *s* and flight position j,1≤j≤ m. Let *E* be the available energy for sensor *s* and A(s)=(b,e] be the set of available slots to sensor *s*. Figure 2 illustrates the SEN-TS-1 problem, and it can be formulated as the following optimization problem:

(19) $$\begin{array}{c} \max\sum_{j=b+1}^{e}log\left(1+\frac{p_{j}}{d_{j}^{\alpha }}\right) \end{array}$$

(20) $$\begin{array}{c} \mathrm{subject}\;\mathrm{to}\sum_{j=b+1}^{e}p_{j}\leq E, \end{array}$$

(21) $$\begin{array}{c} 0\leq p_{j}\leq p^{max},j\in \left(b,e\right]. \end{array}$$

The variables to be determined in these formulations are $p_{j}$, for b+1≤j≤e.

We associate Lagrangian multipliers μ, $\lambda_{j}$, and $\omega_{j}$ with Equations (20)–(21), respectively. Then, the Lagrangian function [36] can be defined as

(22) $$L\left(p,\mu ,\lambda ,\omega \right)=-\sum_{j=b+1}^{e}\log\left(1+\frac{p_{j}}{d_{j}^{\alpha }}\right)+\mu \left(\sum_{j=b+1}^{e}p_{j}-E\right)-\sum_{j=b+1}^{e}\lambda_{j}p_{j}+\sum_{j=b+1}^{e}\omega_{j}\left(p_{j}-p^{max}\right)$$

where p,λ,ω denote the set of $\left\{p_{j}\right\},\left\{\lambda_{j}\right\}$, and $\left\{\omega_{j}\right\}$, b+1≤j≤e, respectively.

Now, in addition to Equations (20)–(21), the necessary and sufficient KKT conditions are:

(23) $$\begin{array}{c} \lambda_{j}p_{j}=0,\forall j\in \left(b,e\right) \end{array}$$

(24) $$\begin{array}{c} \lambda_{j}\geq 0,\forall j\in \left(b,e\right) \end{array}$$

(25) $$\begin{array}{c} \omega_{j}\left(p_{j}-p^{max}\right)=0,\forall j\in \left(b,e\right) \end{array}$$

(26) $$\begin{array}{c} \omega_{j}\geq 0,\forall j\in \left(b,e\right) \end{array}$$

(27) $$\begin{array}{c} \mu (\sum_{j=b+1}^{e}p_{j}-E)=0, \end{array}$$

(28) $$\begin{array}{c} \mu \geq 0, \end{array}$$

(29) $$\begin{array}{c} \frac{\partial L}{\partial p_{j}}=\frac{-1/\ln2}{d_{j}^{\alpha }+p_{j}}+\mu -\lambda_{j}+\omega_{j}=0,\forall j\in \left(b,e\right). \end{array}$$

In the following, we analyze this set of KKT conditions. From Equation (29), we have

(30) $$p_{j}+d_{j}^{\alpha }=\frac{1}{(\mu +\omega_{j}-\lambda_{j})\ln2},\forall j\in \left(b,e\right].$$

According to Equations (21), (23), and (24), there are three possible combinations of the value ranges for $\lambda_{j}$ and $p_{j}$, as Table 1a shows. Similarly, in Equations (21), (25), and (26), there are three possible value range combinations for $\omega_{j}$ and $p_{j}$, as Table 1b shows. Combining Table 1a,b, we obtain all the value range combinations for $\lambda_{j}$, $p_{j}$, and $\omega_{j}$, as Table 1c shows.

From Table 1c, we can see that there are three possible cases for the values of $p_{j}$, $\omega_{j}$ and $\lambda_{j}$. Let us carefully exam Equation (30) for these three cases in the following value assignments for $p_{j}+d_{j}^{\alpha }$:

(31) $$p_{j}+d_{j}^{\alpha }=\left\{\begin{array}{cc} d_{j}^{\alpha }, & \mathrm{if}\;\upsilon \leq d_{j}^{\alpha }\\ \upsilon , & \mathrm{if}\;d_{j}^{\alpha }<\upsilon <p^{\max}+d_{j}^{\alpha },\forall j\in \left[b,e\right]\\ p^{max}+d_{j}^{\alpha }, & \mathrm{if}\;\upsilon \geq p^{\max}+d_{j}^{\alpha } \end{array}\right.$$

where $\upsilon ={\left(\mu \ln2\right)}^{-1}$.

From Equation (31), we obtain an optimality property of the SEN-TS-1 problem, as in Lemma 1.

**Lemma** **1.** *In an optimal solution $p=\{p_{j}|j=b+1,\ldots ,e\}$ to the SEN-TS-1 problem, there exists a unique variable u that satisfies Equation (31).*

**Proof** **of** **Lemma** **1.** It is clear from the aforementioned discussions that, if we know the value of u(>0) for an optimal solution *p*, then we can uniquely determine optimal values of $p=\{p_{j}|j=b+1,\ldots ,e\}$ by Equation (31), because $d_{j}^{\alpha }$ is a known constant. Specifically, $p_{j}+d_{j}^{\alpha }$ equals *u* if *u* is between $d_{j}^{\alpha }$ and $p^{max}+d_{j}^{\alpha }$; otherwise, $p_{j}+d_{j}^{\alpha }$ equals a boundary value, either $d_{j}^{\alpha }$ if $u\leq d_{j}^{\alpha }$, or $p^{max}+d_{j}^{\alpha }$ if $u\geq d_{j}^{\alpha }$. (We can also uniquely determine the value of μ from $u={\left(2\mu \ln2\right)}^{-1}$ directly.) Moreover, the value *u* is unique because the sum of $p_{j},j=b+1,\ldots ,e$ must satisfy $\sum_{j=b+1}^{e}p_{j}=E$ as Equation (20)’s condition. According to Equation (31), $p_{j},j=b+1,\ldots ,e$ monotonically increase with *u*, so there can be only one unique value of *u*. ☐

Thus, the key to solving the SEN-TS-1 problem is to find this unique value of *u*.

### 6.2. The Water-Tank Technique

Here, we introduce the *water-tank* technique, to model and solve the problem of finding the unique value *u*. Note that the deduction of (31) and the *water-tank* technique is inspired by a classic technique: water-filling [36]. However, water-filling cannot handle the constraint $p^{max}$. Besides, *water-tank* is designed as a submodule for the dynamic program (discussed in the next subsection).

**Figure 3:**
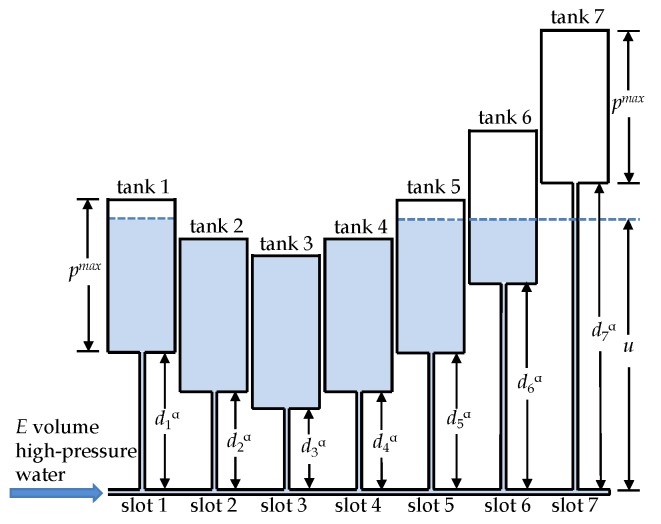
Illustration of the *water-tank* technique. Given a distance set $\left\{d_{j}\right\}$, identical tanks are put at different heights: tank *j* at height $d_{j}^{\alpha }$. Each tank has a unit-area base and a capacity of $p^{max}$. The amount of *E* water is injected into these tanks, and the amount of water in tank *j* is the solution for $p_{j}$.

For each time slot j,b+1≤j≤e, we construct a cylindrical water tank *j* (see Figure 3). All water tanks are identical. Each tank’s vertical length is $p^{max}$ and its base has a unit area so that the capacity of each tank is $p^{max}$. These water tanks are placed at different heights. Specifically, the bottom level of tank *j* is at height $d_{j}^{\alpha }$. Figure 3 shows how every tank is connected by a tube to the horizontal water supply pipe at the ground level, so that water flows freely from tank to tank, but with no leaking to the outside. The pipe has closed and open ends where a valve controls a certain amount of high-pressured water to fill these water tanks.

**Theorem** **1.** *Suppose the described system of water tanks is filled with a total of E volumes of water, excluding the water in the connecting tubes and pipe. Suppose the system’s water level is at height u and the amount of water in tank j is $p_{j},b+1\leq j\leq e$. Then u and $p=\{p_{j}|j=b+1,\ldots ,e\}$ satisfy Equation (31). That is, p is the exact solution for the SEN-TS-1 problem.*

**Proof** **of** **Theorem** **1.** Let us investigate a situation where the system of tanks is filled with a total of *E* volumes of water. Let *u* be the water level as shown in Figure 3 and let $p_{j}$ be the amount of water in tank *j* that equals the height of water from the bottom of tank *j*. Then we have $p_{j}=0$ if $u\leq d_{j}^{\alpha }$; $p_{j}+d_{j}^{\alpha }=u$ if $d_{j}^{\alpha }<u<p^{max}+d_{j}^{\alpha }$; $p_{j}=p^{max}$ if $u\geq p^{max}+d_{j}^{\alpha }$. This matches Equation (31) exactly. Moreover, because the water’s total volume is *E*, we have $\sum_{j=b+1}^{e}p_{j}=E$. Thus, *p* is the solution to the SEN-TS-1 problem. ☐

Algorithm 2 (WaterLevel) presents the pseudocode to design an efficient algorithm to compute water level *u*. (We omit the details because of space limitations.)


**Algorithm 2:**
WaterLevel


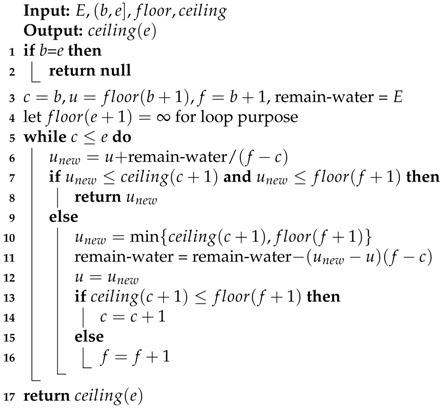



After Algorithm 2 computes the variable *u*, Equation (31) can compute all $p_{j},j\in \left(b,e\right)$. Using WaterLevel as a subroutine, the Algorithm 3 (WaterTank) solves the SEN-TS-1 problem and reports the optimal power control (the transmission rate $p_{j}$) in each slot j∈(b,e], as well as the maximum amount of data collected by the mobile sink. Obviously, WaterTank finishes in O(m) time.


**Algorithm 3:**
WaterTank
  **Input**: E,(b,e],floor,ceiling  **Output**: $\sum_{j=b+1}^{e}\log\left(1+\frac{p_{j}}{d_{j}^{\alpha }}\right)$ and $p_{j}$ **1** Sort tanks by their floor heights; **2** Let $floor\left(j\right)=d_{j}^{\alpha },ceiling\left(j\right)=floor\left(j\right)+p^{max}$, for j∈(b,e]; **3**
u= WATERLEVEL(E,(b,e),floor,ceiling); **4** Compute $p_{j}$ by Equation (31), for j∈(b,e]; **5**
**return**
$\sum_{j=b+1}^{e}\log\left(1+\frac{p_{j}}{d_{j}^{\alpha }}\right)$ and $p_{j}$

**Observation 4**. From Figure 3’s water tank model, or Equation (31), we observe that the tank with smaller $d_{j}$ has a larger value $p_{j}$ in the optimal solution, meaning that it is more productive and energy efficient for the mobile sink to collect data in those slots when it is closer to the sensor.

In the dynamic programming invocation, we may change an available slot from (b,e] to (x,y]⊆(b,e]. Then, we can call WaterTank using (x,y] as the input parameter to find optimal power control for sensor *s* such that the maximum amount of data is collected in slots of (x,y] within energy budget *E*.

### 6.3. Optimally Solving the General SEN-TS Problem

Next, we consider the general SEN-TS problem that involves *n* sensors. According to the definition for SEN-TS problem in Definition 3, we need to assign a set of consecutive slots to each sensor and determine the transmission power for each slot so that the data are collected maximally. Because each slot is assigned to at most one sensor, the problem becomes determining interval $\left(\beta_{i},\varepsilon_{i}\right]$ for sensor $s_{i},1\leq i\leq n$, such that $0\leq \beta_{1}\leq \varepsilon_{1}\leq \beta_{2}\leq \varepsilon_{2}\leq \ldots \beta_{n}\leq \varepsilon_{n}\leq$ m, and the total amount of data transmitted is maximized. Once we determine the transmission interval $\left\{\left(\beta_{i},\varepsilon_{i}\right]\right\}$, we call WaterTank to compute the transmission power (and hence, the transmission rates). Now the problem is how to determine $\left\{\left(\beta_{i},\varepsilon_{i}\right]\right\}$.

We enlarge each interval $\left(\beta_{i},\varepsilon_{i}\right]$ such that the starting point is equal to the ending point $\varepsilon_{i-1}$. The interval $\left(\beta_{i},\varepsilon_{i}\right]$ is enlarged to be $\left(\varepsilon_{i-1},\varepsilon_{i}\right]$, and $\left(\varepsilon_{i-1},\varepsilon_{i}\right]$ may be different from $A(s_{i})$. When this occurs, only slots in $A(s_{i})$ can be used. Thus, the interval of $\left(x,y\right]=\left(\varepsilon_{i-1},\varepsilon_{i}\right]\cap A\left(s_{i}\right)$ is actually used as input for WaterTank. All other slots $\left(\varepsilon_{i-1},\varepsilon_{i}\right]-\left(x,y\right]$, are assigned with zero transmission power. Therefore, the SEN-TS problem’s key task is to find n−1 integers, $0\leq \varepsilon_{1}\leq \varepsilon_{2}\leq \ldots \leq \varepsilon_{n-1}\leq$ m, such that $\left(0,\varepsilon_{1}\right]$ is assigned to $s_{1},\left(\varepsilon_{1},\varepsilon_{2}\right]$ is assigned to $s_{2},\ldots$, $\left(\varepsilon_{n-1},m\right]$ is assigned to $s_{n}$. These integers are called separation points because they separate time into *n* disjoint intervals, one for each sensor. We use dynamic programming to optimally determine all the separation points.

Consider the subproblem that optimally assigns the first *j* slots to the first *i* sensors. Let B(i,j) be the maximum amount of data that the mobile sink *S* can collect from sensors $\left\{s_{1},s_{2},\ldots ,s_{i}\right\}$ in $\left\{slot_{1},slot_{2},\ldots ,slot_{j}\right\}$. Then, our ultimate goal is to compute B(n,m). Let (k,j] be the set of slots assigned to sensor *i* in an optimal solution for B(i,j). Then we have the following relation:

(32) $$B\left(i,j\right)=B\left(i-1,k\right)+\mathrm{WaterTank}(E_{i},\left\{d_{ih}\right\},\left(k,j\right],p_{i}^{max}),$$

where h∈(k,j]. Because 0≤k≤j, dynamic programming can be used to find *k* that maximizes B(i,j). Therefore, the following inductive formula is used for dynamic programming:

(33) $$\left\{\begin{array}{cc} B\left(i,j\right)=\max_{0\leq k\leq j}\left\{B\left(i-1,k\right)+\mathrm{WaterTank}\left(E_{i},\left\{d_{ih}\right\},\left(k,j\right],p_{i}^{max}\right)\right\} & \mathrm{if}\;i>0,j>0\\ B(i,j)=0 & \mathrm{if}\;i=0\;\mathrm{or}\;j=0 \end{array}\right.$$

A pseudocode for the dynamic program is presented in Algorithm 4 (SEN-TS-SVR), which solves the general SEN-TS problem.

**Algorithm 4:** SEN-TS-SVR

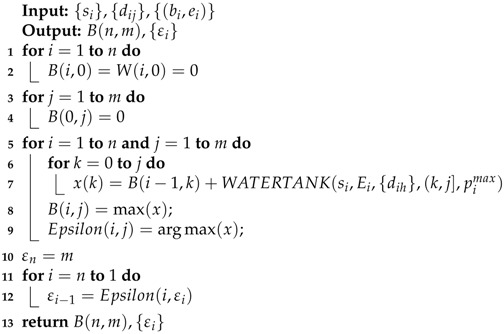



## 7. Evaluations

In this section, we evaluate the proposed algorithm’s performance (named DroneTank) by comparing it against the optimal solution.

### 7.1. Brute Force Searching for the Optimal Solution

Although we have designed algorithms to optimally solve the UAV-FP and SEN-TS problem, respectively, decoupling the joint problem into two subproblems might introduce errors. This subsection introduces a brute force search method to find the optimal solution for the joint problem. Later, we evaluate our proposed algorithms by comparing them to optimal solutions.

In Section 6, we showed that the *water-tank* technique together with dynamic programming optimally solves sensor transmission scheduling for a given flight plan. However, our proposed flight planning might not be optimal if decoupling the joint problem introduces an error. Thus, in our simulation, we employ brute force search for optimal turning points.

More specifically, we enumerate each possible combination of the turning points—namely, the flight plan. Because there are *n* candidate turning-point locations $S_{i},i=1,2,\ldots ,n$, and the UAV selects them in the sequence of their indices, the enumeration is not hard to design. For each enumerated flight plan, if the UAV’s energy budget is not violated, we invoke the transmission schedule procedure to compute the data collection. We choose the flight plan with the largest data collection. The following pseudocode (Algorithm 5) details this approach.


**Algorithm 5:**
BruteForceSearchForOptimal


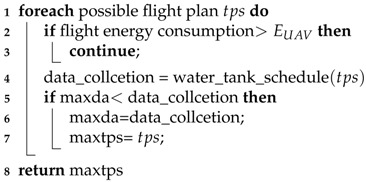



### 7.2. Simulation Settings

We assume the system time is equally slotted, with a slot length 1s. The drone travels at a constant cruise speed of *v* meters per second (m/s), where *v* is set between 7 m/s and 19 m/s in our simulation. To make a turn, the UAV first reduces its speed to stop, and then speeds up to *v* m/s after turning. The slow down and speed up processes each cover 7 m distance and costs 3 seconds. The drone is allowed to make 0-degree turns. Each unit of distance covered by the drone costs one unit of drone energy, and each turn costs an additional *C* units of drone energy, because of slowing and speeding, where *C* is set between 17 and 23 units of drone energy in our simulation. The UAV flies at a height of 5 m to collect data from sensors deployed on the ground.

In our simulations, we consider a set of 11–29 sensors randomly deployed along a smooth-curve natural landscape or rural industrial linear infrastructure, with a total length of 200 m. Brute force searching does not handle large-scale networks efficiently, so we chose to simulate on a small network, to compare it with the optimal solution. During a trip, a sensor can transmit its data to the aerial sink when it is within the transmission range of *d* m, where *d* is set between 9 m and 21 m in our simulation. Between two data collection trips, each sensor harvests a random amount of energy, which follows a uniform distribution U(1,h) mJ, where *h* ranges from 200 to 800. During transmission, the rate *r* and power *p* follow the relation $r=\log(1+p/d^{2})$, where *d* is the distance between the aerial sink and transmitting sensor. The maximum transmission power $p^{max}$ is set to be 330 mW for each sensor.

We use the flight position when the aerial sink is at the slot boundary, to represent its position during such a slot. Thus, we can compute the distance between sensors and flight positions for a given flight plan.

We vary the network size, the aerial sink energy budget, the average sensor energy budget, UAV speed, wireless transmission range, and turn energy cost, one at a time, to study their impacts on algorithm performance.

### 7.3. Simulation Results

**Figure 4:**
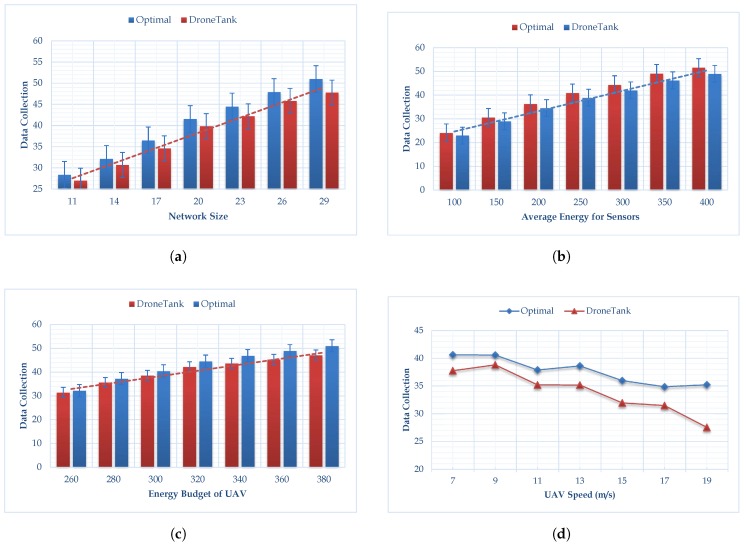

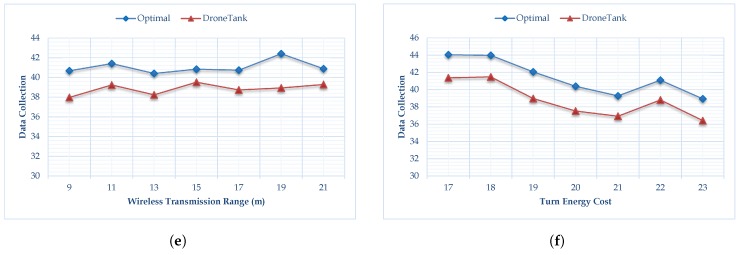
The performance comparisons measured by the total amount of data collected. The default setting parameters are: network size is 20 (number of sensors), the average sensor energy budget is 250 mJ, the UAV energy budget is 300 units, the UAV speed is 7 m/s, the transmission range is 15 m, and the energy cost for each turn is 20 unit UAV energy. (**a**) Impact of network size, varying from 11 to 29, with step of 3. (**b**) Impact of average sensor energy budget, varying from 100 mJ to 400 mJ with step of 50 mJ. (**c**) Impact of UAV energy budget, varying from 260 to 380 with step of 20 units of UAV energy. (**d**) Impact of UAV speed, varying from 7 m/s to 19 m/s, with step 2 m/s. (**e**) Impact of wireless transmission range, varying from 9 m to 21 m, with step 2 m. (**f**) Impact of turn energy cost, varying from 17 to 23, with step 1 unit.

Figure 4 shows the performance comparisons of our proposed algorithm (named DroneTank) to the brute force search algorithm, measured by the total amount of data collected by the mobile sink in one trip. Figure 4a–f show, respectively, how the six parameters affect performance, namely, the number of sensors, the average sensor energy budget for transmission, the UAV energy budget for flight, UAV speed, wireless transmission range, and turn energy cost. Figure 4a shows the results of the simulations, in which we change the number of sensors deployed from 11 to 29 with step 3. We observe that the data collection produced by our algorithm is near the optimal maximum data-collection produced by brute force search. Our proposed algorithm constantly achieves 95 percent of the optimal value, and the performance is quite stable regardless of network size. Figure 4b shows the simulation result when we change the average sensor energy budget for data transmission from 100 to 400 mJ with steps of 50 mJ. Under different average sensor energy budgets, our proposed algorithm performance remains stable, it achieves 95 percent of the optimal data transmission. Moreover, the larger the average sensor energy budget, the larger the data collection by the drone. From Figure 4c, we observe that when we change the UAV energy budget from 260 to 380 with a step of 20 units, our algorithm’s performance drops from around 97 to around 92 percent. This is because the more budget UAV has for flight planning, the more distance the UAV is allowed to cover, and the more turns it is allowed to make. Our proposed algorithm may miss the optimal solution among so many choices. However, the computation time of the brute force search algorithm increases exponentially with the increasing of the UAV energy budget. Figure 4d indicates the data collection is affected by the UAV speed: the higher speed, the less data collected. More specifically, when the UAV speed increases from 7 m/s to 19 m/s with step 2 m/s, the data collection drops for both Optimal and DroneTank. In Figure 4e, we can see the wireless transmission rate does not have much impact on the algorithm performance, partially because the dynamical programming schedules sensor transmission when UAV is close. From Figure 4f, it can be see, if the energy consumption for each turn increases, then less data can be collected by the UAV. This is because, more energy is used in making turns, less energy can be used in covering distance which support energy efficient data collection. Compared to Figure 4a,b,e,f, we can see that the UAV energy budget and the UAV speed have a greater impact on algorithm performance than network size, the average sensor energy budget, transmission range and turn energy cost.

Thus, Figure 4 clearly shows that DroneTank is nearly optimal in variance simulation settings. It is not difficult to explain: although we separate the UAV flight planning from sensor transmission scheduling, our proposed UAV flight planning follows three important observations to find an efficient, optimal solution.

## 8. Conclusions and Future Work

In this paper, we considered a joint problem surrounding UAV flight planning and sensor transmission scheduling for data collection. Our goal was to maximize the data collected by the UAV from sensors, in keeping with energy budget constraints for planning an optimal flight and scheduling efficient sensor transmission. To achieve this, we divided the joint problem into two related subproblems. We then designed optimal algorithms that solve the two problems. Simulations show that our decoupling and solution to the original joint problem are efficient.

There are several interesting directions for future works. First, this paper assumes the line-of-sight wireless channel mode for communication, while in the future, we plan to adopt more sophisticated channel mode, such as air-to-ground channel mode, to characterize more practical scenarios. Second, the propulsion energy consumption for UAV is rather simple, and we plan to model more accurate multi-rotor UAV propulsion energy consumption power on various speeds and on various turning angles based on real-world measurements, so that we can improve the performance of trajectory optimization for UAV to collect data more efficient. Third, we will try to extend our research to a multi-UAV system for other applications in the future when we can fully master the fundamental principles of the single UAV system. Finally, we have assumed 2D flight planning that requires a UAV to fly at a fixed altitude. Such work has certain limitations in practical applications, and we plan to derive the optimal altitude enabling UAVs to achieve a maximum coverage radius for energy-efficient flight planning as another future work.

## Figures and Tables

**Table 1:** Possible Combinations of Value Ranges among Lagrangian Multipliers. (**a**) $\lambda_{j}$ and $p_{j}$; (**b**) $\omega_{j}$ and $p_{j}$; (**c**) $p_{j}$, $\lambda_{j}$ and $\omega_{j}$.

	(**a**)		(**b**)		(**c**)		
Case	$\lambda_{j}$	$p_{j}$	$\omega_{j}$	$p_{j}$	$p_{j}$	$\lambda_{j}$	$\omega_{j}$
1	0	>0	>0	$p^{max}$	=0	≥0	0
2	>0	0	0	$\left[0,p^{max}\right)$	$\in (0,p^{max})$	0	0
3	0	0	0	$p^{max}$	=$p^{max}$	0	≥0
